# Benign Structures Mimicking Right Atrial Masses on Prenatal Ultrasound

**DOI:** 10.1155/2021/8889941

**Published:** 2021-01-12

**Authors:** Snigdha Bhatia, Amna Qasim, Amyn K. Jiwani, Ashraf M. Aly

**Affiliations:** ^1^Department of Pediatrics, University of Texas Medical Branch, Galveston, TX, USA; ^2^Division of Pediatric Cardiology, Texas Children's Hospital, Houston, TX, USA; ^3^Division of Pediatric Cardiology, Department of Pediatrics, University of Texas Medical Branch, Galveston, TX, USA

## Abstract

Advances in imaging have resulted in more frequent reporting of primitive right atrial structures which can sometimes mimic cardiac tumors in prenatal ultrasound. Prominent crista terminalis and Chiari network are examples of these structures. We describe two cases of pregnant women referred to the fetal cardiology clinic for fetal echocardiography for right atrial masses seen on prenatal ultrasound suspicious of tuberous sclerosis. The first case subsequently diagnosed as crista terminalis and the second case as a prominent Chiari network. Postnatal ECHO confirmed the benign nature of these structures. It is important to differentiate tumors from prominent benign structures in the right atrium in fetal ECHO. The location and the similar echogenicity to the adjacent atrial tissue are clues for differentiation of these structures from atrial tumors.

## 1. Introduction

Certain embryonic structures that are present in the fetal right atrium preferentially direct highly oxygenated blood coming from the umbilical veins and the inferior vena cava through the foramen ovale to the left atrium. Advances in imaging have resulted in more frequent reporting of these primitive structures. They can sometimes appear so prominently on prenatal ultrasound that they can be confused with cardiac tumors. Accordingly, a cascade of unwarranted anxiety, further testing, and follow-up may be done for an otherwise benign diagnosis. We report two recent cases that were referred to our fetal cardiology clinic because the prenatal ultrasound was concerning for right atrial tumors.

## 2. Case 1

**Figure 1:**
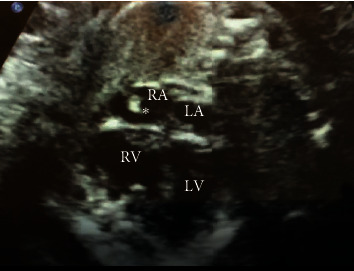
A 4-chamber fetal ECHO showing a 3.4 × 4.6 mm echogenic structure (*∗*) in right atrium (RA) with consistency similar to the adjacent atrial tissue suggestive of crista terminalis.

A 21-year-old Caucasian woman, who was gravida 2 para 0, was noted to have an echogenic mass in the fetal right atrium suspicious for rhabdomyoma on the 23-week prenatal ultrasound. The fetal echocardiogram (ECHO) revealed a 3.4 × 4.6 mm echogenic structure in the right atrium and an otherwise normal cardiac anatomy and function. The mass appeared to have the same consistency as the neighboring atrial tissues (Figure 1). The location of the mass in the upper part of the right atrium was suggestive of a prominent crista terminalis. The mother received prenatal counseling for tuberous sclerosis. The infant underwent serial follow-up with pediatric cardiology for this structure which showed regression, and no further intervention was indicated.

## 3. Case 2

**Figure 2:**
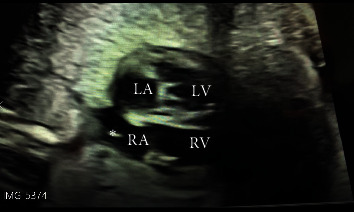
A 4-chamber fetal ECHO showing a 4 × 6 mm echogenic mass in the right atrium near the inferior vena cava suggestive of Chiari network.

A 35-year-old Caucasian woman who was gravida 8 para 5 was referred to the fetal cardiology clinic following the 29-week prenatal ultrasound which showed an echogenic mass in the right atrium. Family history was significant for a daughter born with transposition of the great arteries and ventricular septal defect requiring surgical repair. Fetal ECHO revealed an echogenic oscillating structure measuring 4 × 6 mm in the right atrium near the inferior vena cava prolapsing through the tricuspid valve into the right ventricle and a moderate size membranous ventricular septal defect (Figure 2). The structure in the right atrium was suspected to be a prominent Chiari network. This mother also received prenatal counseling for tuberous sclerosis. The postnatal ECHO confirmed these findings and the baby is being watched for the closing ventricular septal defect.

These two cases are small representations of many pregnant women who are frequently referred to fetal cardiology for suspicion for right atrial tumors.

## 4. Discussion

**Figure 3:**
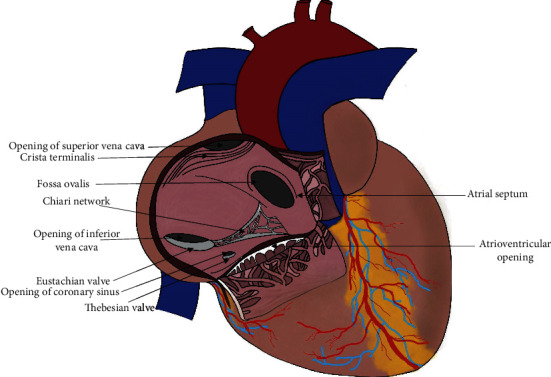
A diagram showing the normal structures in the primitive right atrium.

During embryonic development of the heart, the sinus venosus fuses with the right atrial trabecular appendage. This is followed by regression of the right valve of sinus venosus and the Eustachian valve of the inferior vena cava. Incomplete regression of these structures which support fetal circulation in utero can lead to a spectrum of benign entities such as prominent Chiari network, crista terminalis, Eustachian valve of inferior vena cava, and Thebesian valve of coronary sinus [1] (Figure 3). Review of literature revealed notation of these structures on ECHO, cardiac magnetic resonance imaging (MRI), and autopsy specimens (Table 1).

Crista terminalis is a fibromuscular ridge at the junction of the sinus venosus and the primitive right atrium. It is formed during regression of the septum spurium when the sinus venosus resorbs into the wall of the right atrium. It divides the right atrium into the trabeculated atrium proper and the smooth posterior part. It can be traced from the superior vena cava to the inferior vena cava along the lateral right atrial wall. A prominent crista terminalis is typically seen as a reflective echogenic structure in the right atrium. Careful tracing of the crista terminalis to its origin as well as the use of transesophageal ECHO can help to differentiate it from other cardiac pathologies. Case reports describe the incidental discovery of prominent crista terminalis in adults who underwent ECHO for arrhythmias, dyspnea, or congestive heart failure [4, 8].

The presence of Chiari network is commonly noted on ECHO. It results from the failure of resorption of the right valve of the sinus venosus during fetal development. A Chiari network is typically seen as a “whip-like” reflective echogenic membrane with fenestrations in the right atrium. It can be identified by tracing its attachment to the orifice of the inferior vena cava. Although this may occur as an isolated finding, it can also lead to further cardiac complications such as the formation of thrombi, tricuspid regurgitation, atrial septal aneurysm, and supraventricular arrhythmias [7]. Some cases of Chiari network causing transient fetal hydrops have also been reported [1].

On the other side of the spectrum of right atrial masses lie cardiac tumors and thrombi that can cause fetal or neonatal cardiovascular compromise. Rhabdomyomas are the most common cardiac tumors diagnosed in the fetus and are usually multiple in different chambers of the heart. Isolated right atrial rhabdomyoma is a rare occurrence and can appear as a homogenous hyperechoic mass lesion [9, 10]. An estimated 45–60% of children with tuberous sclerosis have rhabdomyomas [11]. Improved sonographic methods have made the antenatal diagnosis of rhabdomyomas more common in the recent years. The suspicion for such tumors on prenatal ultrasound necessitates further imaging such as fetal ECHO or cardiac MRI and genetic workup. For this reason, it is important to keep in mind normal variants such as crista terminalis and Chiari network to prevent diagnostic confusion and unnecessary testing and anxiety.

## 5. Conclusion

Primitive right atrial structures such as crista terminalis and Chiari network have the same echogenicity as adjacent atrial tissue and have a specific location within the right atrium. Knowledge and experience play an important role in differentiating these anatomical variants from pathological entities.

## Figures and Tables

**Table 1:** Reported normal anatomic variants in the right atrium.

Year of publication	Reference	Number of cases discussed	Structures noted	Imaging modality used	Morphological characteristics
1992	Mirowitz and Gutierrez [2]	20	Crista terminalis Chiari network	Cardiac MRI	Crista terminalis: nodular muscle hypertrophy Chiari network: Muscular ridges
2006	Bhatnagar et al [3]	29	Chiari network	Autopsy specimens	Tissue strands related to the inferior vena cava
2007	Akcay et al [4]	1	Crista terminalis	ECHO	Round, immobile mass in posterior right atrium
2010	Salustri et al [5]	1	Crista terminalis	ECHO, cardiac MRI	Smooth muscular ridge
2012	Obaji et al [6]	1	Chiari network	ECHO	Mobile structure in right atrium
2013	Islam et al [7]	1	Chiari network	ECHO	Whip-like structures extending from the inferior vena cava, moving freely in right atrium

ECHO: echocardiogram; MRI: magnetic resonance imaging.

## Data Availability

All the data supporting this article are included in the article itself

## Abbreviations

ECHO: Echocardiogram
MRI: Magnetic resonance imaging.
